# Self-Assembled 3D ZnO Porous Structures with Exposed Reactive {0001} Facets and Their Enhanced Gas Sensitivity

**DOI:** 10.3390/s130708445

**Published:** 2013-07-02

**Authors:** Jin Chang, Muhammad Z. Ahmad, Wojtek Wlodarski, Eric R. Waclawik

**Affiliations:** 1 School of Chemistry, Physics & Mechanical Engineering, QUT, Brisbane, QLD 4000, Australia; E-Mail: cj4566@gmail.com; 2 School of Electrical & Computer Engineering, RMIT University, Melbourne, VIC 3000, Australia; E-Mails: zamharir@gmail.com (M.Z.A.); ww@rmit.edu.au (W.W.)

**Keywords:** zinc oxide, gas sensors, self-assembly, porous structure

## Abstract

Complex three-dimensional structures comprised of porous ZnO plates were synthesized in a controlled fashion by hydrothermal methods. Through subtle changes to reaction conditions, the ZnO structures could be self-assembled from 20 nm thick nanosheets into grass-like and flower-like structures which led to the exposure of high proportions of ZnO {0001} crystal facets for both these materials. The measured surface area of the flower-like and the grass, or platelet-like ZnO samples were 72.8 and 52.4 m^2^·g^−1^, respectively. Gas sensing results demonstrated that the porous, flower-like ZnO structures exhibited enhanced sensing performance towards NO_2_ gas compared with either grass-like ZnO or commercially sourced ZnO nanoparticle samples. The porous, flower-like ZnO structures provided a high surface area which enhanced the ZnO gas sensor response. X-ray photoelectron spectroscopy characterization revealed that flower-like ZnO samples possessed a higher percentage of oxygen vacancies than the other ZnO sample-types, which also contributed to their excellent gas sensing performance.

## 1. Introduction

Semiconductor gas sensors (SGS) based on metal oxides (e.g., SnO_2_, ZnO, In_2_O_3_, WO_3_, TiO_2_) continue to draw attention due to increased demands for environmental monitoring and protection in both industrial or domestic gas detection settings [1–5]. The interest in SGS arises from advantages such as their compact sizes, their high sensitivities and the possibility of bench fabrication involving comparatively low cost [6]. In principle, the gas-sensing mechanism of metal oxide SGS is based on the change of electrical resistance of the metal oxide thin film when a gas interacts with the crystalline oxide surfaces, particularly at grain boundaries [7]. Several processes, such as oxidation-reduction reactions of the targeted gases, the adsorption of chemical species on the semiconductors, electronic transfer of delocalized conduction-band electrons to localized surface states and vice versa, can all contribute to the sensor's resistance variation [8,9]. The strong dependence of electrical resistance on interfacial effects, grain boundaries and inter-particle contacts in these devices means performance of SGS may be significantly influenced by the size, morphology and surface atomic structures of the gas sensing materials [8,10–13]. For this reason, a particular recent focus of the SGS field has been the synthesis of metal oxides with different morphologies and crystal structures [14,15]. ZnO is a useful test material for studying effects of nanoscale morphology, interfaces and grain-boundary contacts because it can be readily synthesized into a wide range of forms. ZnO nanostructures such as zero-dimensional (0D) quantum dots [16]; one-dimensional (1D) nanorods [17], nanowires [18–20] and nanotubes [21]; two-dimensional (2D) nanosheets [22] and nanobelts [23] and even three-dimensional (3D) nanoflowers [24] and hollow structures [25] *etc.*, can all now be made using either physical deposition or chemical methods. Zinc oxide is an n-type semiconductor that has been widely investigated in both optical and resistive gas sensing [26] and possesses a wide band gap of 3.37 eV and large exciton binding or Rydberg energy of ∼60 meV, which when coupled with its chemical properties makes it a promising material suitable for use in SGS and other applications. Examples of devices where ZnO nanomaterials are used include solar cells [27], photocatalysis [28], in piezoelectric transducers [29], light emitting diodes [30] and field effect transistors [31]. The component ZnO nanostructures' dimensions and morphologies can strongly influence their optoelectronic and catalytic properties in all these applications [32]. Another factor which can sometimes be overlooked is the advantage in device performance which can be gained by increasing the porosity of the active layer, particularly where interface-effects drive device response, such as with SGS. In the case of ZnO, porous 3D structures assembled from 0D, 1D and 2D nanostructure components exhibit potentially useful properties that combine the features of the micrometer and nanometer scale building blocks. These porous structures have an increased number of surface-active sites compared to other forms of ZnO that could favour gas diffusion and mass transport processes and may therefore enhance gas sensing properties [33,34].

It is no surprise then that synthesis of crystalline materials into forms that expose a large percentage of certain facets is being pursued by many researchers, especially in applications that involve surface interactions, because the properties and activities of crystalline materials are very sensitive to surface atomic structures. For example, anatase TiO_2_ crystals with exposed {001} facets have exhibited enhanced energy conversion efficiency in dye-sensitized solar cell applications [35]. ZnO particles with exposed and reactive {0001} facets synthesized by wet-chemical methods have also demonstrated improved photocatalytic activity compared to other ZnO forms [28]. For example, the photo-degradation activity of ZnO nanocrystals was recently investigated by our group, where we observed that the relative activity of the ZnO facets decreased in the order of {10-11} ≫ {0001}, {10-10} [36]. Compared to research into these effects with solar cells and metal oxide photocatalysts, the effects arising through the tailoring of metal oxide crystals to expose particular active facets has been investigated in gas sensors to a far lesser extent [12,37]. In this work, ZnO porous structures with flower-like and platelet-like morphologies where reactive {0001} facets were exposed to the environment were synthesized by a hydrothermal method. Heat-treatment of a precursor template made of basic zinc carbonate and the effects of precursor concentrations and ratios on final morphology were used to the control shape and structure of ZnO forms. Gas sensor testing demonstrated that flower-like ZnO porous structures could exhibit superior gas sensing performance toward the target gas NO_2_ compared to nanoparticle-type SGS. The enhancement in ZnO-nanoflower SGS response could be attributed to the high porosity of the material, combined with what is effectively an active site concentration-effect arising through reactivity of the ZnO {0001} facets toward the target gas.

## 2. Experimental Section

### 2.1. Synthesis of 3D ZnO Porous Structures

All chemicals used in this study were of analytical reagent grade and were all purchased from Sigma-Aldrich Co. Ltd. (Sydney, Australia) The ZnO 3D porous structures were prepared by a two-step procedure: (i) the synthesis of basic zinc carbonate (BZC), and (ii) the thermal decomposition of BZC. For the synthesis of flower-like structures, 1 mmol (0.3 g) of Zn(NO_3_)_2_·6H_2_O was dissolved in 30 mL 0.5 M urea aqueous solution. After sonication, the mixed solution was transferred into a 50 mL Teflon-lined autoclave and kept at 90 °C for 12 h. After reaction, the reactor was cooled down to room temperature naturally. The white precipitate was filtered and rinsed with DI water several times. The obtained precursor was dried in vacuum oven at 60 °C overnight, and then heated at 400 °C for 4 h to obtain the final products. To elucidate the growth mechanisms, two different sets of reactions were mainly carried out for the preparation of hierarchical ZnO structures (details in Table 1). In the first set, the molar ratio of urea to Zn^2+^ precursor was set to 15:1 with [Zn^2+^] = 0.008 M, 0.016 M, 0.033 M and 0.066 M, where the corresponding products were labeled F1, F2, F3 and F4, respectively. In the second set, [Zn^2+^] was set as 0.033 M; and products prepared with different urea/Zn^2+^ molar ratio were labeled F5, F6, F7 and F3 for 1, 5, 10 and 15, respectively.

### 2.2. Characterization

X-ray diffraction spectrometry [XRD, PANanalytical XPert Pro Multi Purpose Diffractometer with Cu *Kα* (*λ* = 0.154178 nm) radiation] was used to determine the structure of the as-prepared samples. The morphologies of hierarchical structures were investigated by field-emission scanning electron microscopy (FE-SEM, JEOL JSM7001F) and transmission electron microscopy (TEM, JEOL JEM2100). Additionally, high-resolution transmission electron microscopy (HR-TEM) images and selected area electron diffraction (SAED) patterns were obtained to determine the crystal structures. The samples for TEM measurements were prepared by placing a drop of ethanolic ZnO solution on a carbon-coated copper grid. Surface areas of the samples were determined from nitrogen adsorption-desorption isotherms at liquid nitrogen temperature using a surface area analyzer (ASAP 2020). The Brunauer-Emmett-Teller (BET) method was used for the surface area calculation and the pore size distribution was estimated by the Barrett-Joyner-Halenda (BJH) method. X-ray photoelectron spectroscopy (XPS) measurements were performed by a Kratos Axis Ultra photoelectron spectrometer incorporating a 165 mm hemispherical electron-energy analyzer.

### 2.3. Sensor Fabrication and Gas Sensing Test

To examine the gas sensing properties of as-prepared ZnO porous structures, sensors constructed from flower-like F3 and grass/platelet-like F5 were tested for response with respect to NO_2_ and compared with a commercial particulate ZnO sample as well. The gas sensor devices were fabricated by spin-coating 1 mg·mL^−1^ ethanolic ZnO solutions onto a SiO_2_ substrate (8 × 12 mm) containing pre-patterned gold interdigitated transducers (IDT). After spin-coating, the samples were dried at room temperature and annealed at 400 °C for 12 h to eliminate any possible occurrence of organic contaminants. For the gas sensing tests, the electrodes were mounted onto a heater inside a chamber equipped with gas-flow manifold and a mass flow controller. The concentration of NO_2_ gas was tuned by changing the ratio of the flow of NO_2_ stream with respect to that of a zero-grade air system. The sensor response, *S*, was defined by *S* = *R_g_*/*R*_air_, where *R_g_* and *R*_air_ were the resistances of the films in the test gas and in zero-grade air gas, respectively.

## 3. Results and Discussion

**Figure 1.**
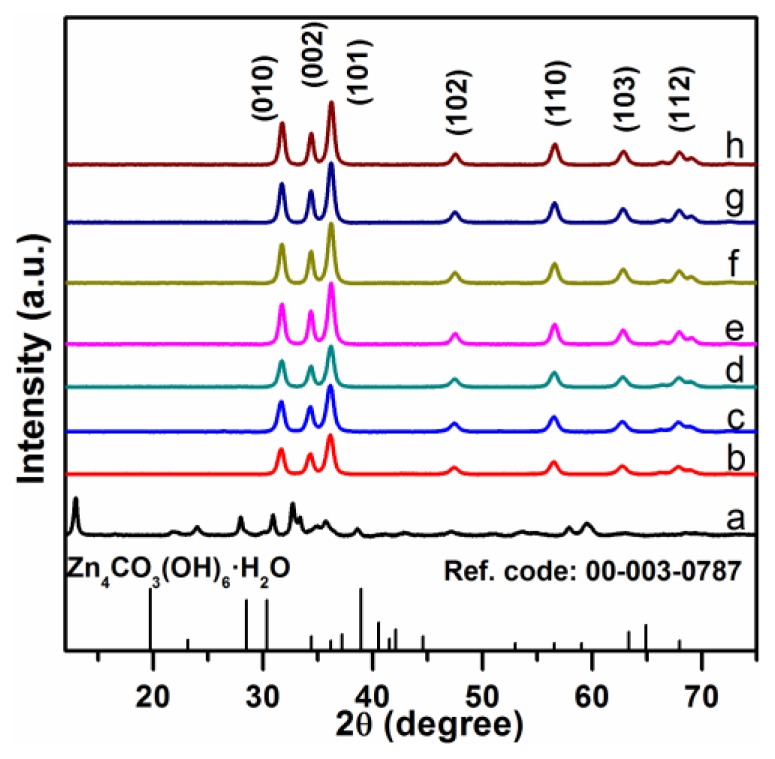
XRD patterns of (a) Zn_5_(CO_3_)_2_(OH)_6_ precursor and (b-h) ZnO nanostructures F1-F7.

Figure 1 shows the X-ray diffraction (XRD) patterns of the precursor and the final products prepared from different [Zn^2+^] concentrations and urea/Zn^2+^ molar ratios. The XRD pattern of the precursor in Figure 1(a) could be unambiguously indexed to the Zn_5_(CO_3_)_2_(OH)_6_ (JCPDS Card No. 11-0287). After annealing at 400 °C for 4 h, the precursor had completely transformed into ZnO, confirmed by XRD patterns of sample F1-F7. The XRD pattern of the final products were indexed as pure Wurtzite ZnO (JCPDS No. 36-1451) with lattice constants of *a* = 0.3244 nm and *c* = 0.5198 nm.

**Figure 2.**
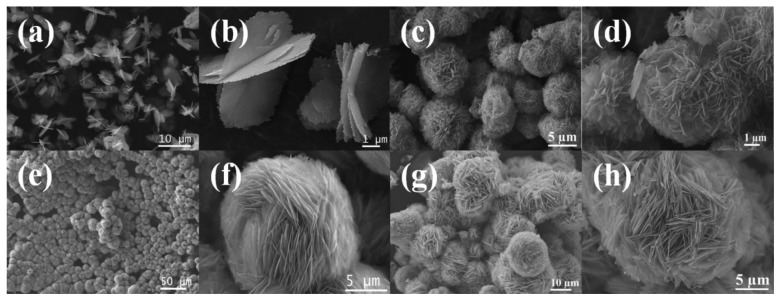
SEM images of ZnO samples F1-F4 prepared with different Zn^2+^ concentrations: (**a**, **b**) 0.008 M; (**c**, **d**) 0.016 M; (**e**, **f**) 0.033 M and (**g**, **h**) 0.066 M.

The morphologies of ZnO structures, prepared under different conditions according to Table 1, were characterized using FE-SEM. The surface areas and XRD-derived crystallite sizes of these ZnO samples are shown in Table 1. Figure 2(a,b) shows the FE-SEM images of F1 prepared with a molar ratio of [urea]/[Zn^2+^] = 15 and 0.008 M of Zn(NO_3_)_2_·6H_2_O. The image clearly shows the formation of three-dimensional ZnO platelet-based structures, where the platelets, or nanosheets intersected along a common line. The structures appeared to be formed from thin blocks (average thickness ∼20 nm) with rough surfaces and jagged edges (Figure S1). The morphologies of samples F2, F3 and F4 prepared with Zn^2+^ concentration of 0.016 M, 0.033 M and 0.066 M were similar except for a slight difference in their compactness (Figure 2(c-h)). Consequently these samples developed a flower-like morphology assembled from the 2D ZnO nanosheet structures. The ZnO structures obviously compacted tighter into shapes reminiscent of flowers when the reactant concentration was increased. It should be noted that the morphology of F4 became less uniform than the samples prepared at lower concentration, which is probably due to faster crystal growth kinetics at high reactant concentration. The average sizes of these flower-like ZnO microstructures changed from approximately 5 to 20 μm when the reactant concentration was increased in the order of F1, F2, F3 and F4.

**Figure 3.**
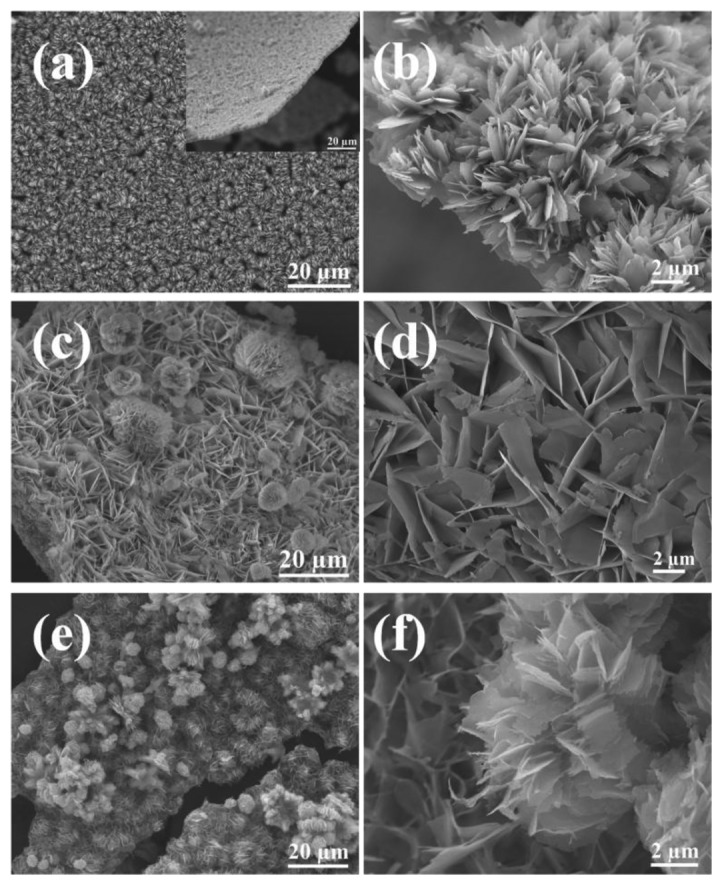
SEM images of ZnO samples F5-F7 prepared with different urea/Zn^2+^ molar ratio R: (**a**,**b**) R = 1; (**c**,**d**) R = 5 and (**e**,**f**) R = 10.

To investigate the effects of the urea/Zn^2+^ molar ratio on the morphology of ZnO products, samples were prepared with different urea/Zn^2+^ molar ratios (*R*) and then examined by FE-SEM. Here, the amount of Zn(NO_3_)_2_·6H_2_O was fixed at 1 mmol, while urea concentration was varied at 1, 5, 10 and 15 mmol. Figure 3(a,b) show the FE-SEM images of sample F5 prepared at the molar ratio of [urea]/[Zn^2+^] = 1. It is shown that sample F5 consists of relatively uniform grass-like, 2D nanosheet structures. These building blocks were similar to that of samples F2, F3 or F4 but for the self-assembly of flower-like morphologies. The morphologies of sample F6 and F7 prepared at the molar ratio of [urea]/[Zn^2+^] = 5 and 10, respectively, were shown in Figure 3(c-f). It was demonstrated that flower-like ZnO structures “bloomed” slowly as the [urea]/[Zn^2+^] molar ratio increased. The grass-like ZnO structure completely changed from 2D plate, into flower-like morphology, when the [urea]/[Zn^2+^] molar ratio was increased to 15, as shown in Figure 2(e,f). Thus, it can be concluded that the [urea]/[Zn^2+^] molar ratio determines the overall morphology of ZnO samples, either grass-like or flower-like; while the concentration of reactants determines the compactness of the hierarchical structures as displayed in Figure 2.

**Figure 4.**
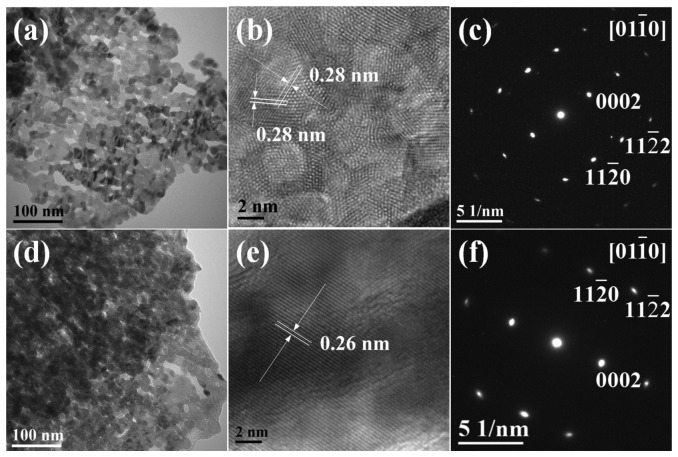
(**a**,**d**) TEM images, (**b**,**e**) HR-TEM images and (**c**,**f**) SAED patterns of ZnO samples F3 and F5, respectively.

To further reveal the structure of as-prepared ZnO samples, TEM, HR-TEM and SAED characterizations were carried out for the representative samples F3 and F5. Figure 4(a) shows a typical TEM image of the building block that underlies these structures, *i.e.*, the nanosheet elements of the ZnO nanoflowers F3. It was revealed that the nanosheets were porous, which is consistent with the rough surface observed during FE-SEM characterization. The HR-TEM (Figure 4(b)) shows that the surfaces of the well-crystallized nanosheets consisted of ZnO nanocrystal aggregates. The 2D lattice fringe spacing was measured to be 0.28 nm, with an angle of around 60°, corresponding to the {10-10} crystal planes of wurtzite ZnO. This observation identified the building blocks of sample F3 as nanosheets with {0001} crystal facets. The SAED pattern (Figure 4(c)) highlights the single-crystalline nature of the porous ZnO sample. Similarly, TEM image of the building blocks of F5 sample also shows evidence for a porous structure (Figure 4(d)). The HR-TEM (Figure 4(e)) gave the lattice fringe with spacing of 0.26 nm, in agreement with the interspacing of {0002} planes. The SAED pattern confirmed that ZnO sample F5 was single-crystalline in nature.

**Figure 5.**
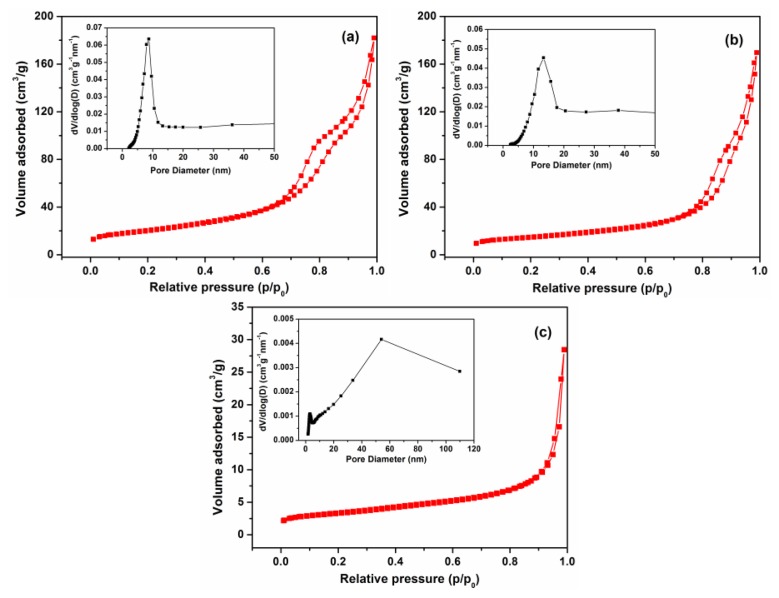
Typical N_2_ gas adsorption-desorption isotherm of the F3, F5 and commercial ZnO samples. Insets: the corresponding pore-size distribution.

Nitrogen adsorption/desorption isotherms and BJH pore diameter measurements of F3, F5 and commercial ZnO samples are given in Figure 5. These isotherms could be categorized as type IV, with a distinct hysteresis loop observed in the range of 0.5-1.0 *p*/*p*_0_, indicating the presence of mesopores (2-50 nm). The BET surface area of F3 was 72.8 m^2^·g^−1^, which is larger than that of F5 (54.8 m^2^·g^−1^) and significantly higher than the commercial ZnO nanoparticle samples (11.8 m^2^·g^−1^). The pore size distribution of F3 exhibits a strong peak centered at ∼8.5 nm and a narrow distribution revealing a uniformity of pore size occurring within the ZnO nanosheets. The average pore size of F5 is around 13.5 nm, with a relatively broader distribution peak that leads to lower BET surface area compared with F3.

Regarding the formation of platelet and flower-like ZnO structural motifs observed, these are frequently observed in minerals based on metal hydroxide salts like the BZC precursor produced in our synthesis [38–40]. Layered double hydroxide salts with a general formula M*_a_*(OH)*_b_*(X*^c−^*)_(2_*_a−b_*_)_/*c*·nH_2_O (M = Zn^2+^, Co^2+^, Ni^2+^, *etc.*; X = Cl^−^, NO^3−^ and CO_3_^2−^) [41], consist of a brucite-type layered crystal structure of metal hydroxides between which, interlayer anions are located that maintain overall charge neutrality. These layers naturally form flower-like structures under high pH conditions prevalent in our study. On the basis of growth modes proposed in [22,42], the ZnO nanostructure formation involves the following sequence of reactions in the aqueous solution containing Zn^2+^ and urea:

(1) $$\text{CO}{\left({\text{NH}}_{2}\right)}_{2}+3{\text{H}}_{2}\text{O}\to {\text{CO}}_{2}↑+2{\text{NH}}_{4}\text{OH}$$

(2) $${\text{NH}}_{4}\text{OH}\to {{\text{NH}}_{4}}^{+}+{\text{OH}}^{-}$$

(3) $$5{\text{Zn}}^{2+}+2{\text{CO}}_{2}+6{\text{OH}}^{-}+2{\text{H}}_{2}\text{O}\to {\text{Zn}}_{5}{\left({\text{CO}}_{3}\right)}_{2}{\left(\text{OH}\right)}_{6}+4{\text{H}}^{+}$$

(4) $${\text{Zn}}_{5}{\left({\text{CO}}_{3}\right)}_{2}{\left(\text{OH}\right)}_{6}\to 5\text{ZnO}+2{\text{CO}}_{2}+3{\text{H}}_{2}\text{O}$$

**Figure 6.**
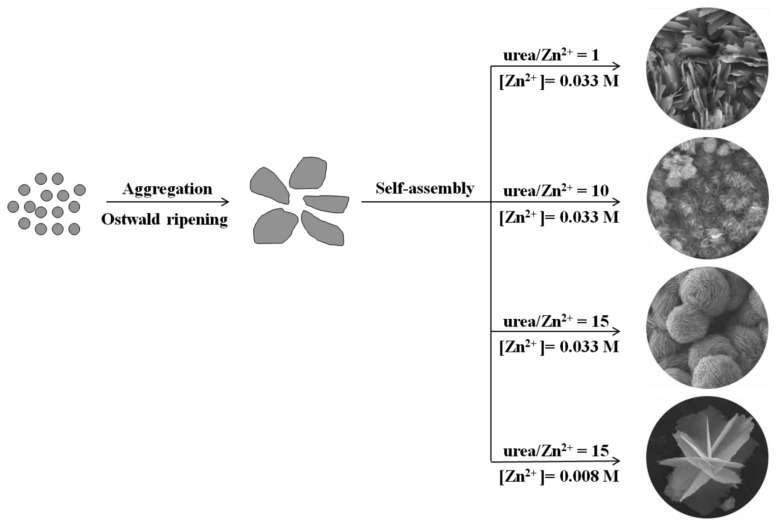
Typical N_2_ gas adsorption-desorption isotherm of the F3, F5 and commercial ZnO samples. Insets: the corresponding pore-size distribution.

When the precursor solution is heated at 90 °C, urea releases CO_2_ and OH^−^, as described in Equations (1) and (2). The CO_2_ is then free to react with Zn^2+^ in the alkali solution and form basic zinc carbonate (Equation (3)). Under annealing at 400 °C, basic zinc carbonate decomposes to release CO_2_ and H_2_O and generates the porous ZnO nanosheets according to Equation (4). For the formation of different morphologies, we consider these to result from the combined effects of Ostwald ripening and self-assembly processes. A possible growth scheme is illustrated in Figure 6, where at first, numerous tiny BZC crystalline nuclei form in the presence of zinc salt in the urea solution at 90 °C and the auto-generated vapor pressure. The BZC nuclei grow through Ostwald ripening and aggregate into layered nanosheet structures. With different precursor concentration and ratio, the crystalline nuclei self-assemble into 3D structures, driven by different growth rates specific for particular crystallographic orientations, generating ZnO with the same morphology after calcinations.

**Figure 7.**
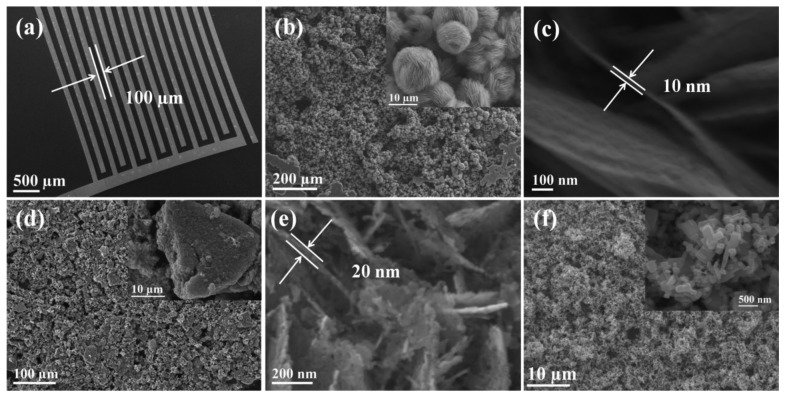
(**a**) SEM image of the pattern of gold interdigitated transducers on a SiO_2_ substrate; (**b**) low-magnification SEM image and enlarged image (inset) of the ZnO-F3 film on gold IDT; (**c**) high-magnification SEM image of the ZnO-F3 sample; (**d**) low-magnification SEM image and enlarged image (inset) of the ZnO-F5 film on gold IDT; (**e**) high-magnification SEM image of the ZnO-F5 sample; (**f**) low-magnification SEM image and enlarged image (inset) of the film of commercial ZnO nanoparticles on gold IDT.

To investigate the gas sensing properties of as prepared ZnO samples, the paste of ZnO-F3, ZnO-F5, and commercial ZnO nanoparticles were prepared in ethanol and separately spin-coated on a SiO_2_ substrate (8 × 12 mm) containing pre-patterned gold IDT. The SEM image of gold IDT is shown in Figure 7(a), which shows that the space between the two neighboring electrodes in the interdigitated pattern is around 100 μm. Figure 7(b) shows the low-magnification SEM image of ZnO-F3 film, which fully covered the gold IDT pattern. The enlarged SEM image is shown in the inset, which shows that the flower morphology of ZnO-F3 was kept during the film preparation process. The thickness of sensing film was estimated to be around 20 μm because the substrate was just covered by ZnO flowers, which were around 10 μm diameter. The high-magnification SEM image in Figure 7(c) shows that the thickness of the plate-like crystallites forming flower-like ZnO-F3 was around 10 nm. SEM characterization for the grass-like ZnO-F5 film on gold IDT pattern indicated that the grass-like morphology of as-prepared sample was destroyed during the paste preparation process [Figure 7(d)]. The inset of Figure 7(d) exhibits that ZnO-F5 nanostructure was broken and aggregated into irregularly shaped bulks. The high-magnification SEM image in Figure 7(e) indicated that thickness of the plate-like crystallites forming ZnO-F5 was around 20 nm, which was thicker than that of ZnO-F3 sample. SEM characterization was also conducted for the commercial ZnO nanoparticle film, which was shown in Figure 7(f). Enlarged SEM image in the inset of Figure 7(f) shows that the particle size was around 200 nm, which was much larger than the individual nanocrystals existed in ZnO-F3 and ZnO-F5 samples.

**Figure 8.**
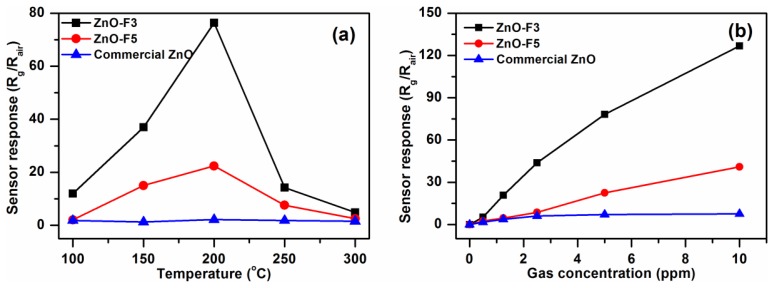
(**a**) Sensor response of sample F3, F5 and commercial ZnO towards NO_2_ of 5 ppm with increasing operating temperatures; (**b**) Gas sensor response of F3, F5 and commercial ZnO to different concentration of NO_2_ at their corresponding optimized operating temperatures.

**Figure 9.**
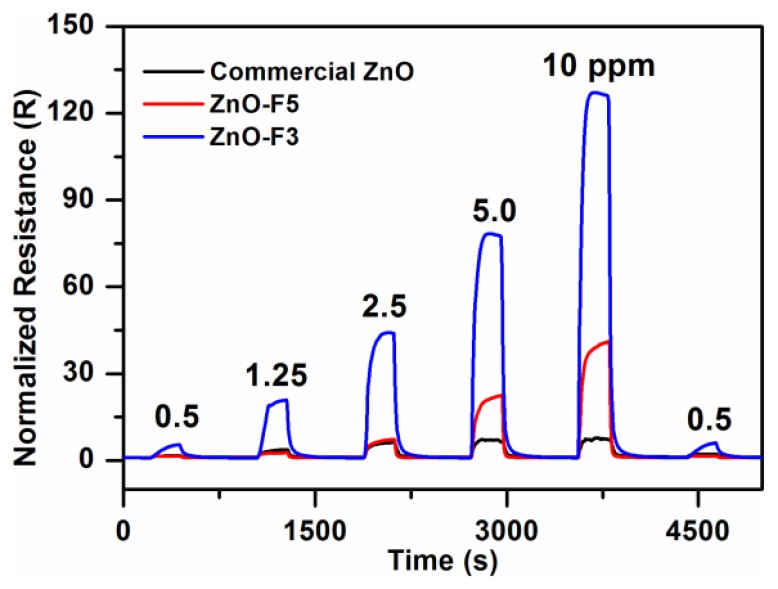
Dynamic response curve of the sensor prepared from F3, F5 and commercial ZnO samples to NO_2_ with concentrations ranging from 0.5 ppm to 10 ppm.

It is well known that the working temperature has a significant effect on the gas sensing response of semiconductor gas sensors. This is due to temperature-dependent adsorption-desorption kinetic processes. To study the gas sensing properties of the flower-like F3 and grass-like F5 porous ZnO structures, a series of experiments were carried out by varying sensor operating temperatures and gas concentrations. Results were compared to commercial ZnO-based sensors. Figure 8(a) shows the responses of the three ZnO samples exposed towards nitrogen dioxide at temperatures between 100°C and 300°C. The sensor responses increased between 100 °C and 200 °C, and then decreased with further increase of temperature. Therefore, the ZnO samples were tested at 200 °C towards different concentrations of NO_2_ gas in the range 0.5–10 ppm. The dynamic response and recovery curves of F3, F5 and commercial ZnO exhibit stable and repeatable response toward NO_2_ gas with short response/recovery times (Figure 9). The response time toward NO_2_ gas of ZnO-F3 and ZnO-F5 films was similar, which was around 60 s. The response amplitude of the three sensors was observed to increase with higher NO_2_ concentration.

As shown in Figure 8(b), the sensor response to increased NO_2_ concentration is not linear, but gradually saturates at about 3 ppm in the case of the commercial ZnO-based SGS. Sensors based on ZnO forms F3 and F5 also displayed this behaviour but to a much lesser extent. This trend would be consistent with a ZnO SGS response where reactions between NO_2_ and a limited number of active sites on the ZnO surfaces occurs, the more porous samples which also happen to possess the greater proportion of high energy exposed ZnO facets might be expected to have a greater number of surface active sites than the commercial nanoparticle ZnO material gram-for-gram. In fact the response of porous structures F3 and F5 are much higher than that of commercial ZnO. For 10 ppm NO_2_, the response of F3 was *ca*. 125, which is about three times that of F5 gas sensor. The high response of F3 might thus be due to the particular flower-like structure with 2D nanoplates, the voids and interspaces existing among nanoplates facilitating gas adsorption and desorption, and the porous structure providing the large surface area with more reactive sites for gas sensing. In addition, by comparing the microstructure of ZnO-F3 and ZnO-F5 sample in Figure 7, it was found that the plate-like building block of ZnO-F3 was thinner than that of ZnO-F5 sample. It was observed that morphology of flower-like ZnO-F3 was kept after film preparation, while the grass-like ZnO-F5 collapsed and aggregated during the paste preparation process. The destruction of grass-like morphology could decrease the contacts between the agglomerated structures of ZnO-F5, resulting in the decrease of gas sensitivity. Compared with destroyed ZnO-F5 nanostructure, the plate-like building block of ZnO-F3 was relative thinner and well contacted with each other for flower-like morphology. Therefore, the sensitivity was likely controlled by the contacts between the agglomerated structures and the thickness of plate-like crystallites.

It is known that the oxygen ions (O_2ads_^−^) would form at the surface of the ZnO nanostructures in a sequence of physisorption and charge exchange reactions with the ZnO crystals, as described by Equation (5). After the ZnO film was exposed to NO_2_ gas, NO_2_ would be chemically adsorbed on ZnO surfaces or react with O_2ads_^−^, via Equation (6), and cause the reduction in the electron concentration, resulting in the increase in the sensor resistance [43,44]:

(5) $${\text{O}}_{2}+{\text{e}}^{-}\to {{\text{O}}_{2}}^{-}(\text{ads})$$

(6) $${\text{NO}}_{2}+{{\text{O}}_{2}}^{-}(\text{ads})+2{\text{e}}^{-}\to \text{N}{{\text{O}}_{2}}^{-}(\text{ads})+2{\text{O}}^{-}(\text{ads})$$

**Figure 10.**
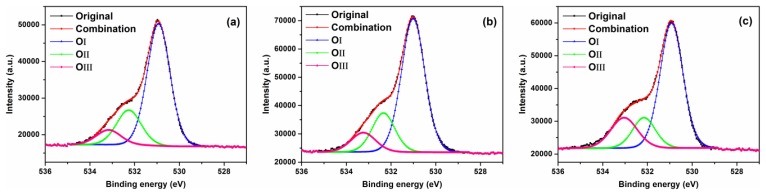
High resolution O1s XPS spectra of the ZnO samples after calcinations: (**a**) flower-like ZnO F3, (**b**) grass-like ZnO F5 and (**c**) commercial ZnO.

In order to investigate the effect of ZnO surface oxygen vacancies on the gas sensing performance of the sensors, the materials were examined using XPS. Figure 10 shows XPS spectra for O1s core level for F3, F5 and commercial ZnO samples. The O1s peak of F3, F5 and commercial ZnO samples on the surface could be consistently fitted by three Gaussian curves, centered at ∼531, ∼532 and ∼533 eV, respectively. The comparison of O1s XPS data for flower-like F3, grass-like F5 and commercial ZnO samples are presented in Table 2. The O_I_ species at the low binding energy of 531 eV belong to O^2−^ ions in the wurtzite ZnO structure [45,46]. The O_II_ species with a medium binding energy centered around 532 eV are attributed to O^−^ and O^2−^ ions in the oxygen deficient regions mainly caused by oxygen vacancies, *V*o [47]. The high binding energy species O_III_ centered at ∼533 eV belong to the absorbed or dissociated oxygen or OH species on the surface of ZnO [47]. As shown in Table 2, the percentage of O_II_ species in F3, F5 and commercial ZnO samples were 21.5%, 19.9% and 16.0%, respectively. Therefore, the flower-like sample F3 has slightly more oxygen vacancies, which are associated with active sites for NO_2_ gas and could contribute to higher gas sensitivity.

## 4. Conclusions

In summary, 3D ZnO porous structures have been synthesized by a hydrothermal method at 90 °C from a basic zinc carbonate precursor, after the thermal decomposition of the basic zinc carbonate template. By properly monitoring the experimental conditions, grass-like and flower-like ZnO porous structures were obtained with surface areas of 52.4 and 72.8 m^2^·g^−1^, respectively. A likely growth mechanism of ZnO hierarchical structures was proposed based on the experimental results. The flower-like and grass-like ZnO gas sensors had different gas sensing properties, with the flower-like SGS exhibiting much higher sensitivity to NO_2_ gas. The nitrogen gas adsorption-desorption isotherm results revealed that the average pore size of flower-like ZnO was smaller than that of grass-like sample, which contributed to higher surface area. The morphology of ZnO-F3 was kept during the paste preparation process, while the morphology of ZnO-F5 was destroyed. The superior gas sensing performance of flower-like ZnO-F3 is likely contributed by the higher surface area and effective contact between the building blocks, which are thin plate-like crystallites. The XPS spectra indicated that the flower-like and grass-like ZnO had a higher ratio of oxygen vacancies than commercial sample, which could contribute to higher sensitivity of ZnO-F3 and ZnO-F5 compared to commercial ZnO nanoparticles.

## Figures and Tables

**Table 1.** Reaction conditions for the preparation of ZnO porous structures and their morphologies.

**Samples**	**[Zn^2+^]/(M)**	**[Urea]/(M)**	**Morphology**	**Surface area**	**Crystallite Size**	**Average size**
F1	0.008	0.120	flower	32.4 m^2^·g**^−^**^1^	13.8 nm	5 μm
F2	0.016	0.240	flower	36.1 m^2^·g**^−^**^1^	13.2 nm	10 μm
F3	0.033	0.495	flower	72.8 m^2^·g**^−^**^1^	14.0 nm	15 μm
F4	0.066	0.990	flower	69.4 m^2^·g**^−^**^1^	16.5 nm	20 μm
F5	0.033	0.033	grass	54.8 m^2^·g**^−^**^1^	14.3 nm	-
F6	0.033	0.165	grass & flower	52.4 m^2^·g**^−^**^1^	17.8 nm	-
F7	0.033	0.330	grass & flower	51.1 m^2^·g**^−^**^1^	21.9 nm	10 μm

**Table 2.** Comparison of O1s XPS data for different ZnO samples.

**ZnO samples**	**Peak O_I_**	**O_I_(%)**	**Peak O_II_**	**O_II_(%)**	**Peak O_III_**	**O_III_(%)**
F3	530.94	69.4	532.29	21.5	533.20	9.10
F5	531.00	69.5	532.34	19.9	533.25	10.6
Commercial	530.93	65.4	532.18	16.0	533.06	18.6

## Supplementary Files

Supplementary File 1 Supporting Information (PDF, 255 KB)
