# Supplementary material for: Self-Assembled 3D ZnO Porous Structures with Exposed Reactive {0001} Facets and Their Enhanced Gas Sensitivity
Source: Sensors (Basel). 2013 Jul 2;13(7):8445–60. doi: 10.3390/s130708445 (PMC3758604; doi:10.3390/s130708445)
Supplement: Supplementary File 1 — Supporting Information (PDF, 255 KB) [file sensors-13-08445-s001.pdf]

OPEN ACCESS

sensors

ISSN 1424-8220
www.mdpi.com/journal/sensors

Supporting Information

Self-Assembled 3D ZnO Porous Structures with Exposed Reactive {0001} Facets and Their Enhanced Gas Sensitivity. *Sensors* 2013, *14*, 8445-8460

Jin Chang [1], Muhammad Z. Ahmad [2], Wojtek Wlodarski [2] and Eric R. Waclawik [1,*]

[1] School of Chemistry, Physics & Mechanical Engineering, QUT, Brisbane, QLD 4000, Australia; E-Mail: cj4566@gmail.com

[2] School of Electrical & Computer Engineering, RMIT University, Melbourne, VIC 3000, Australia; E-Mails: zamharir@gmail.com (M.Z.A.); ww@rmit.edu.au (W.W.)

* Author to whom correspondence should be addressed; E-Mail: e.waclawik@qut.edu.au; Tel.: +61-7-3138-25791; Fax: +61-7-3138-1804.

Figure S1. FE-SEM image of ZnO sample F1.

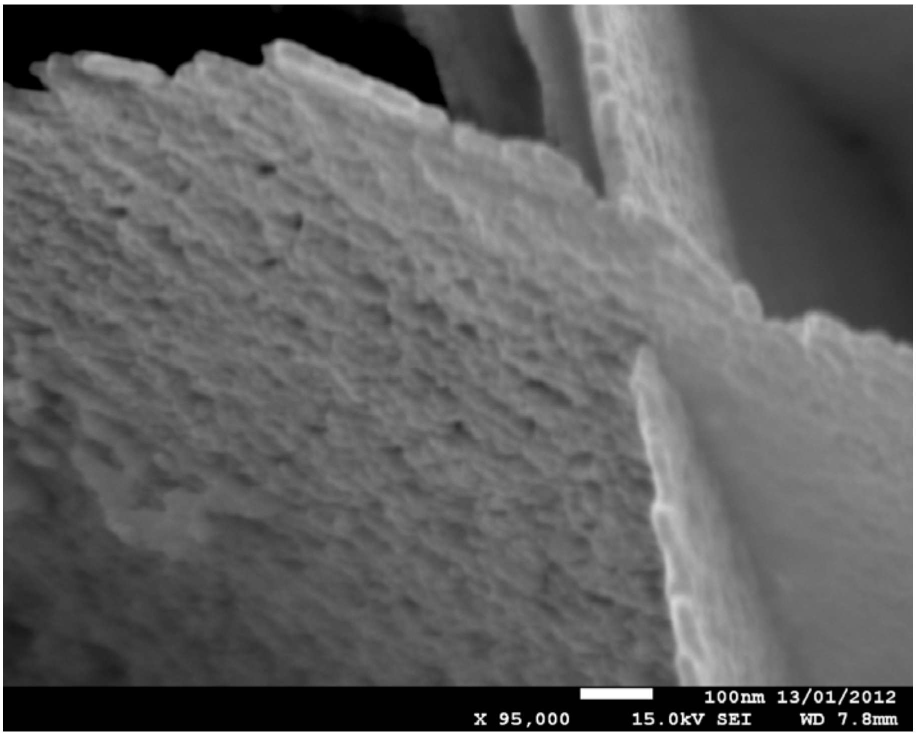

© 2013 by the authors; licensee MDPI, Basel, Switzerland. This article is an open access article distributed under the terms and conditions of the Creative Commons Attribution license (http://creativecommons.org/licenses/by/3.0/).
